# Cost-effective filtering of unreliable proximity detection results based on BLE RSSI and IMU readings using smartphones

**DOI:** 10.1038/s41598-022-06201-y

**Published:** 2022-02-14

**Authors:** Katarzyna Filus, Sławomir Nowak, Joanna Domańska, Jakub Duda

**Affiliations:** 1 Institute of Theoretical and Applied Informatics, Polish Academy of Sciences, Bałtycka 5, Gliwice, 44-100 Poland; 2 Meetlify, Aleksandra Lubomirskiego 27/1, Kraków, 31-509 Poland

**Keywords:** Computational science, Computer science

## Abstract

Indoor environments are a major challenge in the domain of location-based services due to the inability to use GPS. Currently, Bluetooth Low Energy has been the most commonly used technology for such services due to its low cost, low power consumption, ubiquitous availability in smartphones and the dependence of the signal strength on the distance between devices. The article proposes a system that detects the proximity between static (anchors) and moving objects, evaluates the quality of this prediction and filters out the unreliable results based on custom metrics. We define three metrics: two matrics based on RSSI and Intertial Measurement Unit (IMU) readings and one joint metric. This way the filtering is based on both, the external information (RSSI) and the internal information (IMU). To process the IMU data, we use machine learning activity recognition models (we apply feature selection and compare three models and choose the best one—Gradient Boosted Decision Trees). The proposed system is flexible and can be easily customized. The great majority of operations can be conducted directly on smartphones. The solution is easy to implement, cost-efficient and can be deployed in real-life applications (MICE industry, museums, industry).

## Introduction

Indoor environments are a major challenge in the domain of location-based services (LBS). Although, global positioning system (GPS) can offer a very accurate position estimate in the outdoor environments, it is highly inaccurate in the indoor environments due to Radio Frequency signal blocking^1^. Therefore, much effort has been made to create solutions that do not use Global Navigation Satellite System, among others based on Bluetooth^2,3^, ultra-wideband (UWB)^4,5^ and ZigBee^6,7^ and some hybrid approaches (e.g. based on Wi-Fi and Bluetooth Low Energy^8^).

In the recent years, Bluetooth Low Energy (BLE) has been the most commonly used technology for indoor proximity estimation and localization due to its low cost, low power consumption, ubiquitous availability in mobile devices and the dependence of the Received Signal Strength Index (RSSI) on the distance between the broadcast BLE beacons^3,9^. Broadcasting BLE beacons involves periodically transmitting a radio signal containing a predefined message (called Advertisement Frames, denoted as ADV) in the surrounding environment. Such frames can be received from any BLE-enabled device, in particular smartphones. Approaches based on the received signal strength allow a reasonably accurate proximity estimation, especially when beacons are individually calibrated and appropriately distributed in space. This approach is considered the most practical^10^ and the vast majority of indoor distance localization and estimation works developed in recent years are based on this method, with different approximation models and methods used to filter incoming frames.

Although the domain of indoor proximity and location estimation is dominated by approaches using Bluetooth Low Energy RSSI, the Bluetooth signal in indoor environment has low stability and sensitivity to interference (many obstacles, signal reflections etc.)^11^. To minimize these problems sophisticated mathematical methods (e.g. smoothing filters and wavelet filters) are used in the systems proposed in the literature, which make them impractical in situation, in which data for numerous nodes has to be analyzed. Also, when the object is moving, the quality of signal further decreases and from practical point of view, localization and proximity detection may lead to very misleading results^12^.

The problem addressed in this paper concerns tracking the activity of participants in meetings, trade fairs and conferences and is related to the meetings industry (MICE, meeting-incentive-conferences-exhibitions). The work described was conducted as a part of the AIMeet project under the Bridge Alfa program, an initiative of the Polish National Center for Research and Development, focused on commercialization of research projects. Project aims to deliver a solution that provides analytical functions for business meeting organizers. It was assumed that the solution to the problem is not to create an indoor localization system, but to determine for each participant successive, time-ordered interactions with waypoints (in this paper we use the term “static point” to describe these objects) over time. This requires appropriate analysis of sensor signals and radio signal strength (measured as RSSI) to determine the appropriate activities and to indicate the correct moments of interaction and filter the remaining ones. The key problem, which we aim to solve using our system, is to indicate the correct interactions of a participant with a waypoint, and to distinguish between behaviors and interactions that are irrelevant to the design objectives (moving between stands). This requires the development of methods that recognize specific individual behaviors of participants (activities) and specific interactions of participants in relation to objects (waypoints), such as trade fair stands or other distinguished points of interaction. It is important to develop solutions that can be scaled to environments with large numbers of participants (such as exhibitions or trade fairs).

In our system, in order to achieve good performance and minimize the error proneness, we limit the costly operations and incorporate a lightweight model for activity recognition and introduce reliability metrics that allow us to evaluate and filter proximity predictions (proximity of a dynamic and static objects). Our approach to proximity detection is based not only on data from the external world (RSSI from static points—anchors) but also on information about the internal state of the object under study (expressed by the readings from the Inertial Measurement Unit (IMU) of a smartphone). We do not use any benchmark dataset and instead we create our own one, because none of the datasets available online meets all of the necessary requirements: availability of static and dynamic activities, dependence of IMU readings on BLE frames arrivals, availability of raw sensory data, realistic positions of smartphones (e.g. in hand, in a pocket). We use smartphones (which already have all of the necessary hardware functionalities and only a simple application is needed) to gather data from both static and dynamic objects, which makes our approach cheaper and easier to deploy than the one based on dedicated iBeacons and wearable sensors. Despite this cost-efficiency, our approach delivers high-quality and reliable results.

In “Related work” we present a literature review in the domain of indoor localization and proximity recognition, as well as Human Activity Recognition. In “Materials and methods” we describe the methods and data used in our experiments. In “Results” we present the obtained results and further discuss them in “Discussion”. We conclude the article in “Conclusions”.

## Related work

Due to the fact that Global Navigation Satellite Systems (GNSS) and Global Positioning Systems (GPS) which provide accurate location services for outdoor environments cannot be used in the indoor environments to obtain an accurate position due to Radio Frequency signal blocking^1^. Therefore, different technologies are used as a data source for indoor positioning and localization, among others Radio Frequency Identification (RFI)^13^, Ultrasonic Positioning (UIP)^14^, Bluetooth^2^, Ultra-Wideband (UWB) technology^4^ and ZigBee^6^.

Out of these technologies, BLE technology has become the most popular one in the domain of distance and proximity assessment in indoor environments in recent years^9^. The basic approach is curve fitting based on the collected RSSI measurements to find a suitable equation for distance estimation^15^, different approximation models are also used depending on the coarse distance estimation, e.g.^16^.

Also, indoor localization solutions for localization and positioning based on BLE have been developed. The trilateration technique based on RSSI is mostly used. Indoor localization systems, regardless of the technology used, suffer from high position errors. In particular, RSSI for BLE technology is susceptible to noise and multipath effects, which significantly reduces its localization accuracy, and in addition, the LoS (loss of sight) problem has a great impact, especially in crowded rooms and interiors with obstacles^17^. The accuracy of RSSI calculations can be improved by calibration, use of dedicated devices and analysis of radio signal propagation. This approach however entails high hardware costs and requires preparation of the environment, as demonstrated in^18^.

To increase accuracy, localization and positioning solutions are sometimes augmented with data from additional sensors (e.g.^18–20^). In^18^, a complex hybrid localization algorithm is described, consisting of pose and velocity estimation based on activity detection and accelerometer readings and using RSSI signal strength. Despite the precision indicated in the paper, which is higher than the other techniques, the method remains computationally complex and scaling it to a sufficiently large number of users remains a problem. Generally, systems created for the purpose of localization and positioning are based on computationally-demanding methods and could not be deployed in environments, in which data from numerous nodes is analyzed (e.g. Generalized Regression Neural Network and Kalman filter in^21^ or Denoising Autoencoders in^3^). A broader review of techniques related to indoor localization is presented in^17^.

However, in applications such as data analysis for logistics applications (e.g. trade fairs, museums, galleries, industry), tracking the trajectories of people and their interactions relative to specific objects is sufficient. In such applications, the problem corresponds to mutual positioning and detection of object and user interactions, rather than location (in the sense of indicating the exact position of objects).

The method of solving the problem of localization, proximity estimation and activity recognition depends on the application area. For the MICE industry, the interactions with specific objects are crucial and the number of visitors can be very large, therefore the approaches should be highly scalable and efficient. We have embedded our area of interest in the field of distance and location estimation research based on BLE signal, therefore in the following part of the section we focus on the work and problems most related to our proposed solution.

In work^22^ a method for detecting the trajectories of museum visitors relative to exhibits is described. Analyzing museum visitor behavior is becoming increasingly important and digital technologies become ubiquitous. The paper describes the analysis of data provided by low-cost mobile devices and stationary proximity sensors to understand museum visitor behavior. Specifically, they address which works of art visitors view during their visit, for how long, and in what order. Such evaluations can help determine the popularity of artworks, optimize the layout of ongoing exhibitions, and improve exhibitions. A similar problem was solved in^23^, in which BLE beacons were used. However, the authors faced some problems related to BLE signal and channel variability, leading to ambiguity in positioning accuracy with respect to objects. Distance-to-object data can also be used to analyze typical user behavior and activity profiles. This type of problem was addressed in the paper^24^, in which groups of visitors representing specific social behavior profiles were extracted. BLE beacons were used, deployed both in the museum halls and provided to the visitors. The method used allowed for an accurate trajectory reconstruction at the expense of appropriate deployment and the use of dedicated sensors.

A separate, though related, problem is activity analysis (HAR, Human Activity Recognition). There are many research papers available in this area. Many approaches and fields of application can be distinguished. As a data source, usually sensors are used. These sensors can belong to the smartphone’s Inertial Measurement Unit (IMU), which are equipped with different types of inertial sensors (e.g., gyroscopes and accelerometers). Smartphones also have the ability (resources) to process signals directly. As an alternative, some applications use dedicated hardware instead of smartphones. For example, in^25^, the authors proposed a system for activity recognition using Naïve Bayes classifier, minimum distance and K-Nearest Neighbor (KNN) classification algorithms. The paper^26^ describes a method for recognizing basic activities, based on smartphone inertial sensors—accelerometer and gyroscope. Deep learning methods were used. The aim was to recognize twelve different physical activities (including standing, sitting, lying down, walking, going up and down stairs). The average recognition rate was 89.61% (accuracy 95.85%). Also, in^27^ Deep Learning model were used (Deep Recurrent Neural Networks with Long Short-Term Memory layers in particular). The results obtained in this study showed that Deep Learning models used outperformed statistical ML models (support vector machine and k-nearest neighbors) and other neural network models: Deep Belief Networks and Convolutional Neural Networks (CNNs). For analysis, they used a dataset with raw body-worn sensors readings. In a different study^28^, the authors used CNNs to extract local features from accelerometer data together with some simple statistical features.

Some benchmark datasets for HAR task have been introduced in the literature, e.g.^29–31^. Dataset^29^ includes six activities from daily living (walking, walking upstairs, downstairs, sitting, standing and laying). During the experiment, people were wearing a smartphone (Samsung Galaxy S II) on the waist. Readings from an accelerometer and a gyroscope were gathered. The readings were preprocessed using noise filters and sampled in fixed-width sliding windows. Butterworth low-pass filter was used to separate body and gravity acceleration. Dataset^30^ consists of data gathered using a high performance IMU (accelerometer and gyroscope) sensor positioned on the right hip of the volunteers. Also, barometer readings were considered. 12 human activities were considered: walking left, right, forward, upstairs, downstairs, running forward, jumping up, sitting, standing, sleeping, riding elevator up and down. For the purpose of^31^, data was gathered from on-body sensors (seven IMUs and twelve accelerometers placed on different body parts). 18 home activities were considered, among others opening and closing doors, opening and closing drawers, opening and closing a fridge and a dishwasher and cleaning a table. The biggest disadvantage of the available datasets is the fact that they involve some specific placement of devices or the use of specialized devices, which makes them impractical for training models used in real life. The alternative to these datasets can be some of the PACT datasets^32^, which consist of Bluetooth RSSI data. Some of the datasets also include smartphone sensory data (from common mobile device positions including handbag, shirt pocket and hand). Unfortunately, the sensory data is gathered independently of Bluetooth frames (therefore it is impossible to combine the data regarding RSSI and sensor readings) and only static activities were considered. Due to the fact that none of the datasets in the literature meets all of the requirements of the methods proposed in the article, dedicated dataset had to be used in the experiments.

## Materials and methods

In this section we present all methods and data used in this work to enable other researchers to replicate the experiments, build the corresponding solution and adjust it to their needs.

### Problem and aim definition

The main objective of the proposed system is to detect the proximity of a moving object with respect to static points (anchors) and to evaluate the quality of this detection. Proximity detection and localization are particularly prone to errors when an object moves between static points (the position is then inherently unstable). In this case, moments of movement should be filtered out. Such filtering gives us additional information about the dynamics of the examined object and can be used to improve the quality of fingerprinting (object tracking).

This formulation of the problem is very general and has many practical applications, including the ones related to MICE industry—e.g. detection of presence at a given stand and analysis of interest regarding some specific static points, in museums and various types of exhibitions—e.g., intelligent recommendations and additional educational materials, but also in Industry 4.0 (e.g., detection of presence of workers at a given machine), or in security applications (e.g., evacuation surveillance). In general, in all situations where we need reliable detection of proximity to some fixed point for analysis and/or execution of appropriate actions.

### System overview

**Figure 1:**
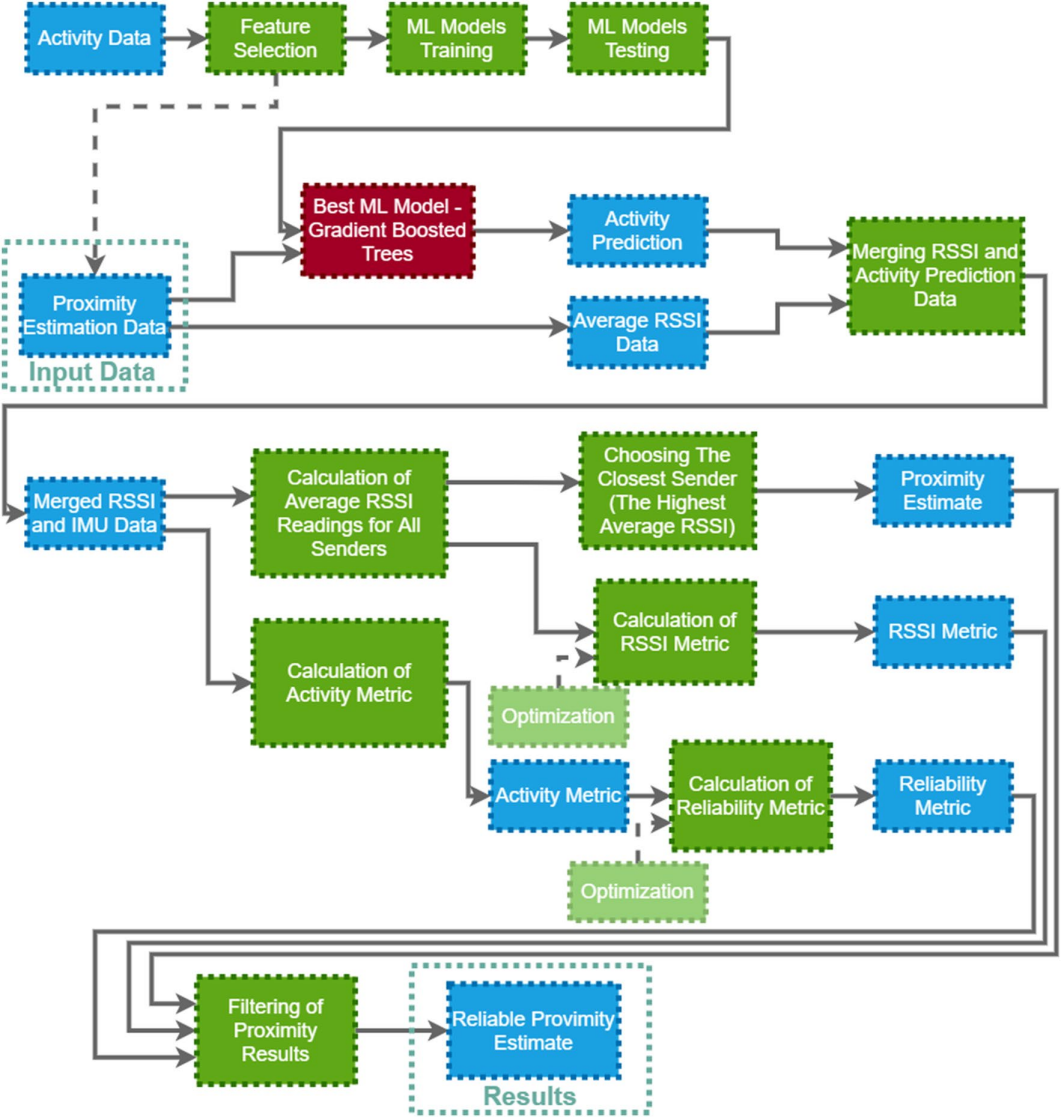
An overview of the system.

As an answer to the problem formulated in “Problem and aim definition”, we propose a system based on Bluetooth technology and IMU readings from smartphones for detecting proximity to a static point (some point of interest) and evaluating the quality of this prediction (based on determining the degree of dynamics of a moving object (e.g., a person) based on introduced metrics obtained based on predictions of a lightweight and efficient machine learning model for activity recognition and Bluetooth RSSI readings from the surrounding static points. The developed solution has been tested in a real environment using smartphones (as anchors and for a moving object), making it a practical and easy to deploy solution. Nevertheless, the presented methodology can also be used in an environment with dedicated Bluetooth beacons. In Fig. 1 we present the overview of the proposed system.

Our analysis consisted of two phases. In phase one, we created and tested various machine learning models for activity recognition, and then chose the best model to use in our system (see “Models”). This model uses the most informative features (these features were determined on the basis of feature selection, see "Feature selection").

In phase two, we processed the input data for the proximity detection task (RSSI readings for the examined static points and the readings from the IMU dependent on the arrival of Bluetooth frames) using the model for activity recognition (only the IMU readings were used) and on their basis we determined the average RSSI values to all static points at a given a moment, and then average values of these readings in a specific time window (in our case about 5s). Based on the predictions from the activity recognition model and the RSSI values, we created basic metrics, and then a joint metric that determines the reliability of proximity estimation in the time window. We determined the coefficients used to calculate the metrics in the optimization process (we manually labelled the data regarding the time windows, in which we examine the proximity (1 for movement, 0 for no movement) and calculated the values of Pearson Correlation coefficient between values of appropriate metrics and true label and chose the values of parameters for which the correlation was the strongest). Ultimately, our system returns information about the nearest static point in an examined time window (proximity detection) and a metric defining the reliability of this proximity estimation, and enables filtering of unreliable estimation results. The proposed method can be modified relatively easily to return the N closest points. Then, using other methods, one can analyze where the dynamic point actually is (in the context of fixed points).

### Activity recognition

In this section, we describe the data, methods, and models we used to create and select an efficient and accurate activity recognition model that we use in the proposed system.

#### Training data gathering and processing

As it was explained earlier, we decided not to use any benchmark datasets, because none of the datasets available online meets all of the necessary requirements: availability of static and dynamic activities, dependence of IMU readings on BLE ADV frames arrivals, availability of raw sensory data. Therefore, to collect the training data, we used an application that simultaneously collects and sends Bluetooth ADV frames from and to other registered devices. When a Bluetooth ADV frame is received, the values returned by the built-in IMU sensors are measured: accelerometer, gyroscope and magnetometer. We use the raw IMU readings to create our training set. Data from these three sensors was also used e.g. in works^33^ and^34^.

We then calculated the values of basic statistical features for each axis of the IMU data, namely mean, standard deviation, minimum value, maximum value, median and skew. Generally, approaches based on time-domain features (mean value, median, variance, skewness, kurtosis, percentiles and interquartile range) are the most popular and proven successful^35^. We decided to use the rolling window of 25 samples (a relatively narrow sliding window for the activity classifier) to determine these values to limit the number of historic data that has to be stored and processed on a smartphone, as our target was to create an economic solution. In our case 25 samples translates to about a quarter of a second—such a time window was also used in work^36^, in which authors obtained accuracy of approximately 90% for HAR based on accelerometer data exclusively for Random Forest algorithm).

We decided to examine four basic activities, which are characteristic for a variety of environments:

- A smartphone lying on the table (884,815 training and testing 3,131,752 samples)
- A person with a smartphone stands (619,666 training and testing 331,502 samples)
- A person with a smartphone slowly walks from one point to another (67,280 training and testing 34,698 samples)
- A person with a smartphone walks fast/runs from one point to another (17,272 training and testing 19,125 samples).

The first two positions represent static activities (they represent no movement of phone/person in space) and the last two ones—the dynamic ones (they represent movement of phone/person in space) in a binary classification task. Due to such a formulation of the problem, the considered binary classification task can be treated as movement detection (for this reason, the class denoting movement is assigned the value 1—the positive one).

We gathered data for different positions of the smartphone, namely smartphone was held in a hand, front and back trousers pockets, in a jacket pocket and in a bag—these are typical positions of smartphones in real-life situations.

As can be seen from the number of samples in each class, the dataset is unbalanced, so we discarded random samples (random undersampling) from the training set for the majority classes and equalized the number of samples with the minority class. We did this because machine learning models are generally sensitive to unbalanced datasets.

Finally, we used the feature selection method described in "Feature selection" and thus selected the most relevant features to create efficient and lightweight models.

#### Feature selection

High-dimensional training feature sets can contain irrelevant information, which impedes the learning process because the relevant information has to be found in this large feature space. Moreover, a large number of features can lead to model overfitting to the training data and insufficient generalization for new data. Feature selection can reduce this issue and contribute to better model generalization. Dimensionality reduction can reduce the time required to train ML models, and allows to create small, light-weight and efficient models, thus models that are fast in prediction due to their smaller size and which can even operate in real time. For these models, the computing resources typical of smartphones are sufficient.

Different techniques can be used for feature selection, generally there are three principal approaches to this task: Filter-Based methods, Wrapper-Based methods and Embedded-Based methods^37^. Wrapper-Based methods and Embedded-Based methods involve using some predictor in the selection process. Wrapper-Based methods operate based on a model that is treated as a “black box” to rank the subsets of training features regarding their predictive power. Embedded-Based methods, on the other hand, choose the best features during the learning process. Filter-Based approaches provide a ranking of all features based on a specific relevance index. As a relevance index, e.g. correlation coefficients can be used (i.e. Pearson, Spearman, Kendall). Correlation-based approaches assess the relevance of the features based on the degree of dependence of the target variable (in a numerical form) on the feature values. Different statistics can be used here, e.g. classical test statistics (Chi-squared, T-test, etc.). Generally Filer-Based methods do not use any predictor performance optimization to select relevant features.

Correlation techniques are often used to determine the relationship between continuous variables. Spearman’s or Kendall’s correlation coefficients indicate the occurrence of monotonic relationships (even non-linear) and are non-parametric. Pearson Correlation coefficient determines the linear relationships and is a parametric measure^38^. Ordinal variables can be treated as continuous ones. Although, using Pearson Correlation with ordinal variables can introduce some potentially incorrect estimation of the relationships, the feature selection methods based on Pearson correlation are robust and can usually successfully find and asses the relationship even when the assumption regarding continuous variables is violated^39^. For that reason, we decided to use Pearson Correlation as a Filter-Based method in the process of feature selection.

To obtain the numerical labels, we encoded the labels in the following manner for binary classification task:

- A smartphone lying on the table/ a person with a smartphone stands (static activities) $\rightarrow$ 0.0
- A person with a smartphone slowly walks from one point to another/ a person with a smartphone walks fast/runs from one point to another (dynamic activities) $\rightarrow$ 1.0.

This representation is the most natural, because in this way we treat the decision variable as the ordinal variable that it actually is (the activities are ranked with respect to the degree of activity).

#### Models

We used three tree-based machine learning models in our experiments to choose the best model for activity recognition. We trained and tested all of these model in a binary classification tasks. We used the following models:

- *Decision Tree* model that relies on classifying data based on a series of responses. The tree is taught to make decisions based on samples from the training set, for which it uses a series of questions (conditions) to determine the membership of a given sample to a particular class. Using the decision algorithm, a root of the tree is created and the data is distributed based on the value of the feature selected using the selection algorithm. The data is then distributed according to this algorithm in the descendant nodes until the leaves of the tree (represented by labels) are obtained.
- *Gradient Boosted Decision Trees* an ensemble classification model that uses decision trees. Boosting involves the use of weak learners (decision trees) and building a single strong learner in an iterativly. Traditional boosted trees scarifies some decision tree’s advantages: e.g. speed and interpretability. Gradient boosted models are a generalization of boosting that was created to mitigate these issues, thus deliver an accurate and effective ML method. Improvement of the classification quality in Gradient Boosted Trees is based on an optimization of a differentiable loss function^40^.
- *Random Forest* an ensemble classification model, an extension of the Bagging method. It is a solution that offers good scalability, classification efficiency and easy application. Random forest is an ensemble of weak classifiers—decision trees trained on subsets of the training set, which combined in an ensemble form a single strong learner. Such a classifier has lower generalization error and lower sensitivity to overfitting. Majority voting is performed to determine the label^40^.

Decision Tree, Gradient Boosted Decision Trees, and Random Forest are three of the most well-known ML algorithms. Decision Tree is a good baseline due to its simplicity. This algorithm was frequently used in the domain of HAR (e.g. in works^41^ or^42^). Ensemble models (Gradient Boosted Decision Trees and Random Forest) gained popularity due to their applicability when dealing with statistical features in numerous diverse fields (e.g. finance^43^ or detecting cardiac abnormalities^44^). They also found application (and they were proven successful) in works considering different types of human activity recognition (e.g. classification of alpine skiing styles based on IMU data^45^ and accelerometer-based human activity recognition in works^46^ or^42^). Due to high accuracy of the described algorithms in the literature, we also decided to use them to build our ML activity recognition models.

We tested these ML models’ performance on test data (from different experiments than those regarding the data gathering for training data for more representative evaluation). We chose the best model based on models’ overall accuracy and accuracy of specific classes prediction of the models (True Positive and True Negative Rates).

### Proximity and reliability estimation

The main goal of the proposed system is to determine the closest static point for the examined dynamic object and filter out the results obtained for the time periods, in which the dynamic object was moving. In this section, we describe the testing environment, the data gathering and processing, as well as the definition of metrics used in the proposed system.

#### Testing environment

**Figure 2:**
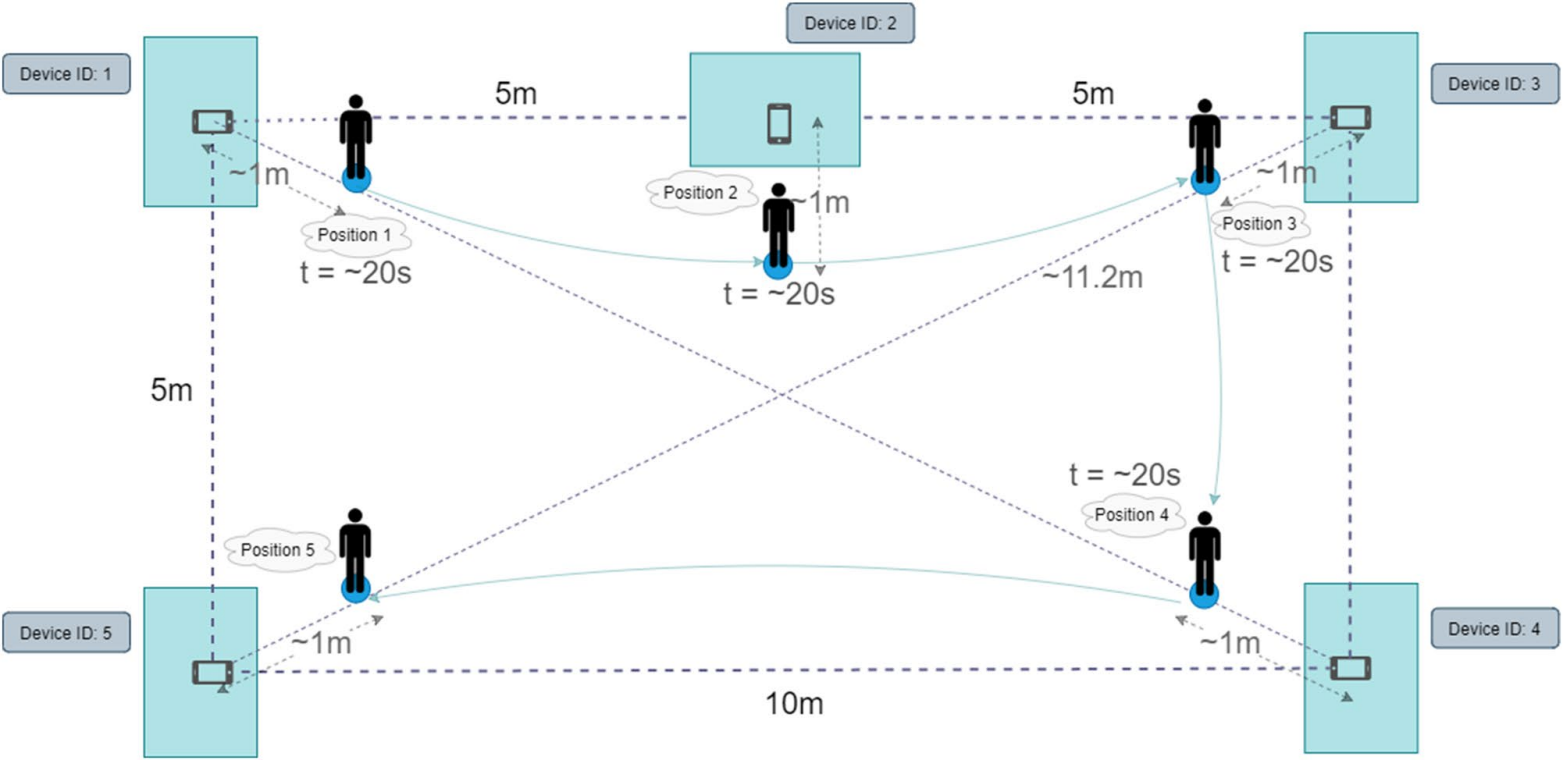
An overview of the experiment.

In order to demonstrate the operation of the proposed system we built a realistic test environment (generalization of a fair hall or a museum hall with stands/art pieces). We also focused on making the experiment procedure itself as realistic as possible. The environment consist of 5 smartphones (static ones) lying on 5 tables, which for the shape of a rectangle (4 smartphones as vertices and the remaining one in the middle of one of the longer edge (see Fig. 2). We numbered the tables in a clockwise manner.

In such a built environment, we placed a person with a smartphone (the experiment lasted two minutes). The person was supposed to behave naturally (as a visitor of a fair hall or a museum hall and watch the stands (static points) and move between them (1 $\rightarrow$ 2 $\rightarrow$ 3 $\rightarrow$ 4 $\rightarrow$ 5) in turns). The person stood at a given stand (starting from the first one) for about 20 seconds, and after a given sign (the second person measured the time) moved to the next stand (it took slightly more than 5 seconds, during the last part—the longest one—the person moved the most rapidly—to make the way last a comparable time as for the other cases, and at the same time to provide a more dynamic case). For this reason, we mark the moments of movement with a margin in the experimental part (2 time windows in the fist two experiments and three in the last two ones—it becomes more difficult to determine the precise ground truth at the end of the experiment).

#### Data gathering

For data collection, we used an application that simultaneously collects and sends Bluetooth ADV frames from and to other registered devices (previously mentioned in “Training data gathering and processing”). When a device receives a Bluetooth ADV frame it measures the values returned by the built-in IMU sensors: accelerometer, gyroscope and magnetometer. Collecting data in a way that depends on the reception of Bluetooth frames, we get RSSI values and sensor readings related to a single timestamp. This property is later used in our experiments. Of course, the app runs on all phones at the same time (start and stop of data collection is scheduled at a specific time—the clocks on the phones are synchronized).

In the experiments, we use only the data received by the phone held by the moving person, and consider phones lying on tables only as transmitters (we treat them as anchors). Naturally, it is possible to use two types of applications—for static phones (lying on tables—anchors) and for dynamic phones (held by people moving inside the examined environment). However, the proposed solution is more practical and allows to use one application both as a transmitter and as a receiver. The application used for measurements was implemented for iOS (the iPhones used were models XR-12).

In the case of Apple smartphones, the BLE operation (e.g. ADV frame sending frequency) is controlled by the IOS operating system, and the details have not been documented in detail^47^. The reference may be the technical information created by and for IOS developers e.g.^48^. The operation depends on several parameters (such as CPU load, memory usage, operating mode, e.g. foreground/background operation). However, to the best of our knowledge, it is not affected by the speed of movement of users.

The measurements in the article were collected in the foreground mode of measuring application, which allowed us to achieve a constant frequency of ADV frames from each device. Of course, it would be possible to create a system with more control over the BLE operation, however it would require using dedicated BLE beacons instead of smartphones, which introduces additional costs and decreases practicality of a solution.

#### Data processing

To estimate the proximity and built the metrics that can be used to assess the reliability of proximity detection, we have to first process raw RSSI and sensor readings to obtain the data necessary to run our algorithms. In Table 1, we present the necessary columns of the data table with the corresponding descriptions.

**Table 1:** Description of columns used in the dataset that can be used for proximity detection compatible with our method.

Column	Description
Timestamp	Timestamp determined for all senders
sender_1	Average RSSI for sender 1
sender_i	Average RSSI for sender i
sender_n	Average RSSI for sender n
Activity prediction	Average value of activity prediction

To build such a table, for each Bluetooth ADV frame we take the corresponding sensor data readings (the same timestamp) and using the features determined in the feature selection process and the trained model, we obtain the predicted activity labels for each Bluetooth frame. We calculate the average value of the label over *n* frames—the final table will be approximately *n* times smaller, because RSSI reading for each sender have to be obtained for particular time moment—this way we obtain more stable values of position estimation over time (it is no longer binary). Then, we create a table, in which we connect the nearest frames for all of the senders (considering timestamp of frame arrival). Such table stores the RSSI readings for sender_1 ... sender_i ... sender_n at some moment. Then, we connect this RSSI table with the determined non-binary label values.

Columns sender_1 ... sender_i ... sender_n represent the average values of RSSI read by the receiver for each static sender (anchor) available in the experiment. We obtain values of average RSSI for each anchor in time window—each time window is approximated by a constant number of readings—rows in a table (depending on the number of Bluetooth frames that are sent by each static sender and the number of static senders—the more senders the more Bluetooth frames we obtain).

In our experiment we have got 5 senders, so to obtain the primary activity label average value, we use the rolling window of length 5. In our experiments we decided to use time windows of length 5 seconds (approximately 100 rows of momentary RSSI readings and primary activity label average values).

#### Metrics definition

We created three metrics to assess the reliability of proximity detection: two basic metrics based on activity recognition results and RSSI (*activity_metric* and *rssi_metric*), as well as one joint reliability metric (*prediction_reliability_metric*).

The ***activity_metric*** can be defined as an average prediction of an activity prediction model in a time window. As we treat the activity prediction task as a binary classification task (raw classifier results are 0 for static activities (smartphone lying on a table, standing) and 1 for dynamic positions (walking, running), such an obtained value is a real number in range $\langle 0; 1 \rangle$. It can be interpreted as a “person movement factor”.

The ***rssi_metric*** is based on absolute changes in RSSI measurements between two consecutive time windows. It can be calculated as follows:

First, let us define $X^{(t)}$ as a vector containing average RSSI readings for all senders (*n*) for time window t, $x_i^{(t)}$ is the average RSSI for *i*-th sender.

Then, the absolute values for each element of the vector are calculated—we obtain vector $|X^{(t)}|$ with elements $|x_i^{(t)}|$.

Let us define $X_{diff}$ as a vector containing the absolute values of differences of average RSSI values between two consecutive time windows:

1 $$\begin{aligned} X_{diff}^{(t)} = \left| X^{(t)} - X^{(t - 1)}\right| \end{aligned}$$

Then, we calculate the weight vector that will be used to calculate the weighed average of changes in average RSSI values between two consecutive time windows. To determine the values of weights for each RSSI difference, we use the *softmax* function:

2 $$\begin{aligned} W^{(t)} = \sigma \left( |X^{(t)}|\right) \end{aligned}$$

To calculate the value of $\sigma (|x_i^{(t)}|)$ the following formula was be used (to obtain bigger values for higher RSSI values, which represent closer static points—characterized by more reliable signal values and potentially bigger changes in the case of position change), the basis of the exponential function has to take values from range (0; 1):

3 $$\begin{aligned} \sigma (|x_i^{(t)}|) = \frac{\beta ^{|x_i^{(t)}|}}{\sum _j \beta ^{|x_j^{(t)}|}}, \quad \beta \in (0; 1) \end{aligned}$$

Then we determine the weighted absolute values of RSSI change ($Y^{(t)}$ with values $y_i^{(t)}$) and calculate the weighted average—$\mu _Y^{(t)}$ (by $*$ we represent multiplication of values at corresponding positions in vectors *W* and $X_{diff}$):

4 $$\begin{aligned} Y^{(t)}= & {} W^{(t)} * X_{diff}^{(t)} \end{aligned}$$

5 $$\begin{aligned} \mu _Y^{(t)}= & {} \frac{\sum _j y_j^{(t)}}{n} \end{aligned}$$

Finally, to obtain the value of metric representing the RSSI change ($rssi\_metric$) for time window *t* we use hyperbolic tangent function, which transforms the metric value to range (0, 1) with smaller values representing smaller changes in RSSI and bigger values—bigger changes (resulting from potential posiotion changes). We use the average value of RSSI change from the next time window (change should be visible after position change):

6 $$\begin{aligned} rssi\_metric^{(t)} = tanh(\mu _Y^{(t + 1)}) \end{aligned}$$

Metric ***prediction_reliability_metric*** is determined based on the values of *activity_metric* and *rssi_metric*, as follows:

7 $$\begin{aligned} prediction\_reliability\_metric^{(t)} = 1 - (\alpha \cdot activity\_metric^{(t)} + \gamma \cdot rssi\_metric^{(t)}), \end{aligned}$$

where $\alpha$ can be described as an importance coefficient of the activity metric and:

8 $$\begin{aligned} \alpha \in \langle 0; 1 \rangle , \quad \gamma \in \langle 0; 1 \rangle , \quad \alpha + \gamma = 1 \end{aligned}$$

As a result we obtain a reliability metric with values in range $\langle 0; 1 \rangle$ with smaller values representing less reliable proximity detection and higher—more reliable estimation - due to potential movement of the person.

All of these metrics can be used to filter out the moving points in the proximity detection task and to assess the reliability of the detection (especially proximity between a dynamic and static point while the dynamic object is moving towards some different location) and are easy to interpret—higher values of *activity_metric* and *rssi_metric* suggest that movement of a person can be involved, whereas the higher values of *prediction_reliability_metric* values suggest that it is more probable that the examined person was moving between points during the time taken into consideration.

## Results

**Figure 3:**
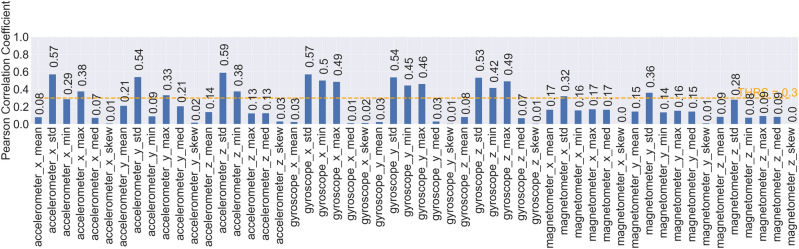
Filter-based Feature Selection based on the absolute value of Pearson Correlation coefficient determined for features and label in a numerical form.

First, we performed the filter-based feature selection based on Pearson Correlation coefficient values. The results show that by taking into consideration only features with the coefficient value greater than 0.3, we were able to minimize the number of features used for training from 54 to 17 (see Fig. 3). The great majority of the rejected features obtained very low values of Pearson correlation coefficient - suggesting a low impact of these features on the label value.

**Figure 4:**
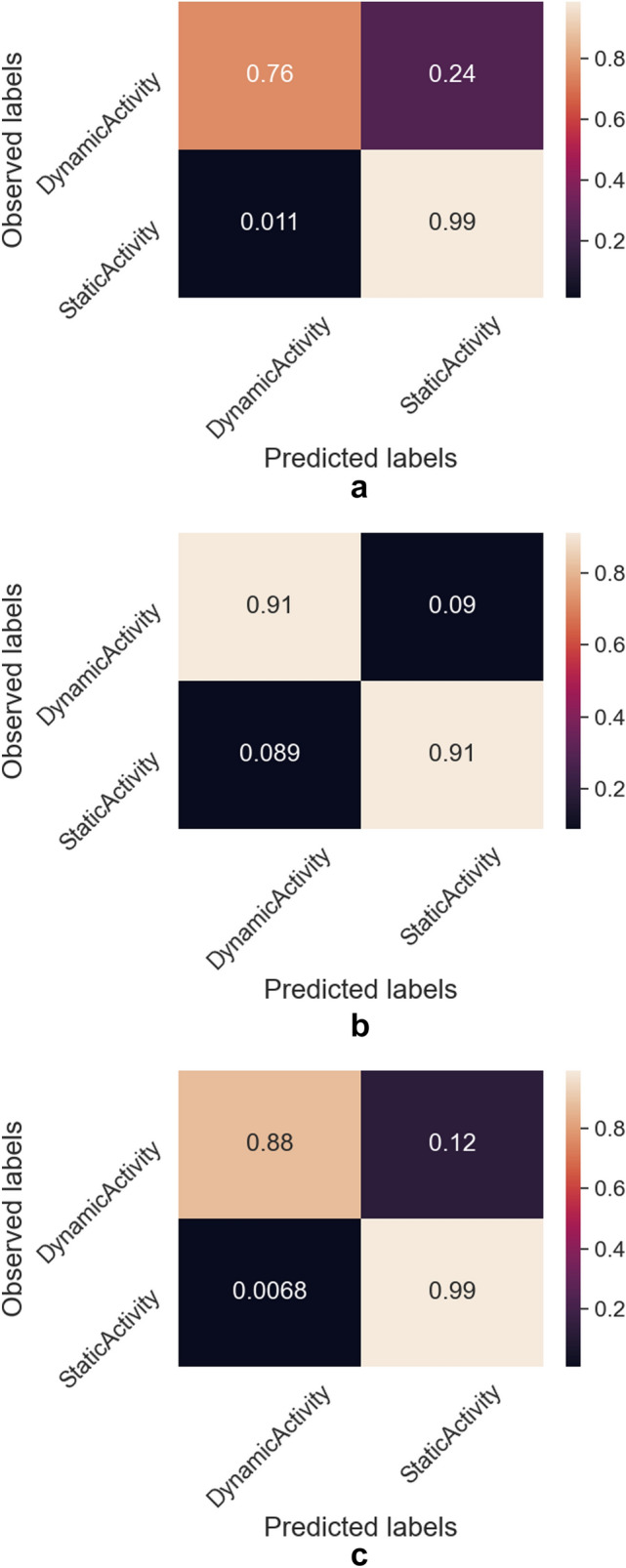
Confusion matrices of examined activity recognition binary models. (**a**) Decision Tree. (**b**) Gradient Boosted Trees. (**c**) Random Forest.

We processed the training dataset and took only the features chosen in the feature selection procedure. We used such a dataset to train three binary models: Decision Tree, Gradient Boosted Decision Trees ensemble model and Random Forest ensemble model. In Fig. 4, we present the performance of the trained models on the test dataset (their confusion matrices). It can be observed that the ensemble models obtained much better results than a traditional Decision Tree. Most probably it is caused by a better prediction power and generalization of the ensemble models. The best performance was obtained by the Gradient Boosted Decision Trees (both True Positive and True Negative rates over 90%), therefore we decided to use this particular model in our further experiments.

**Figure 5:**
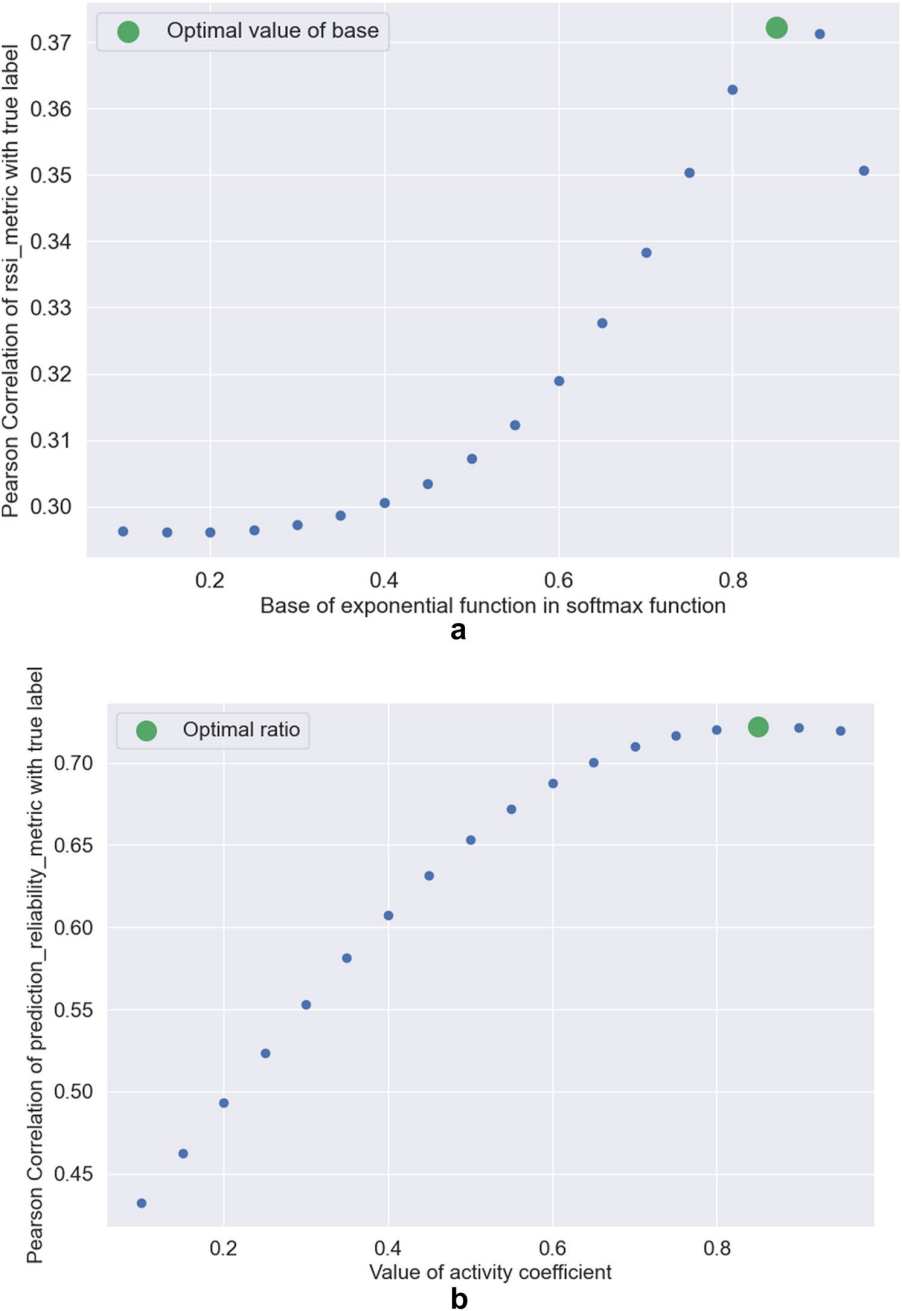
Determining the optimal values of parameters: (**a**) exponent base used in the softmax function; (**b**) importance coefficient used for *activity_metric*.

Then, we processed the data gathered in our testing environment (see Fig. 2): among others, we formatted the data and ran the Gradient Boosted Decision Trees model to obtain the predictions. To find the optimal value of the base in our softmax function (used to calculate the value of a RSSI-based metric), we calculated the values of Pearson Correlation coefficient between values of RSSI-based metric obtained with different values of base (from 0.1 to 0.95 with step 0.05) and chose the base for which the correlation was the strongest—0.85 (see Fig. 5a). Similarly, we chose the optimal value for the importance coefficient (the value for which the correlation between the importance coefficient used for activity metric)—also 0.85. Alternatively, the values can be chosen by the operator (especially the second value—specifying the proportion in which we consider metrics based on RSSI and on activity predictions (see Fig. 5b).

**Figure 6:**
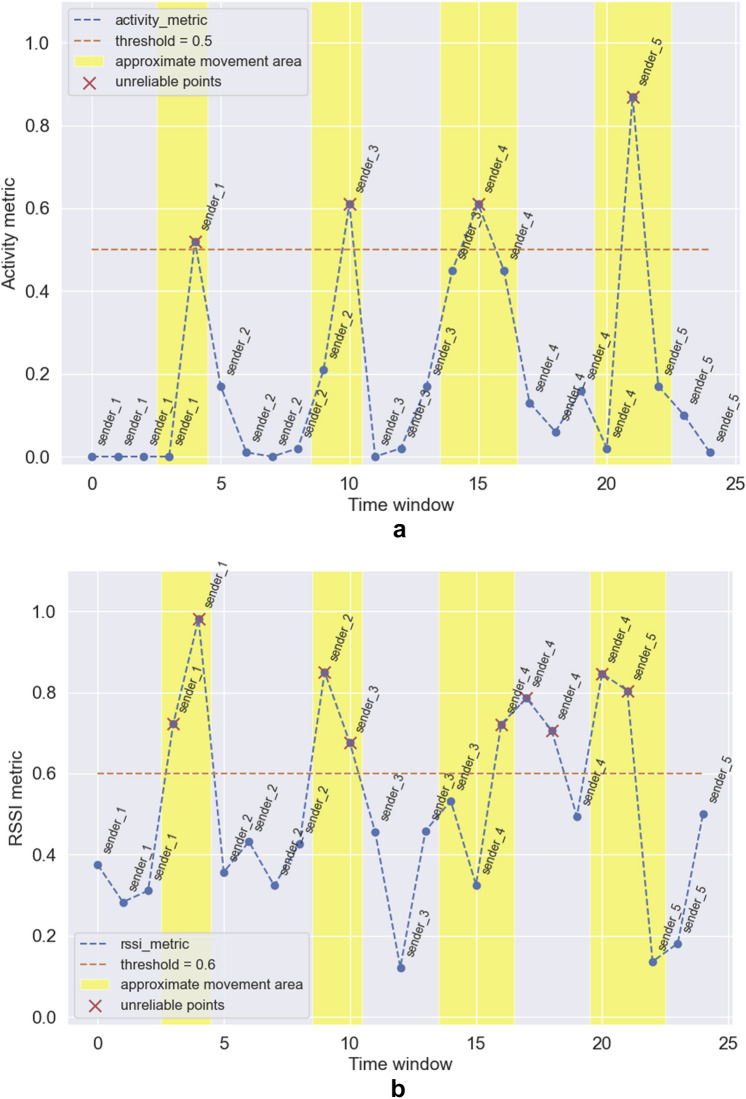
Filtering of unreliable proximity estimation results based on base metrics: (**a**) activity metric; (**b**) RSSI metric.

**Figure 7:**
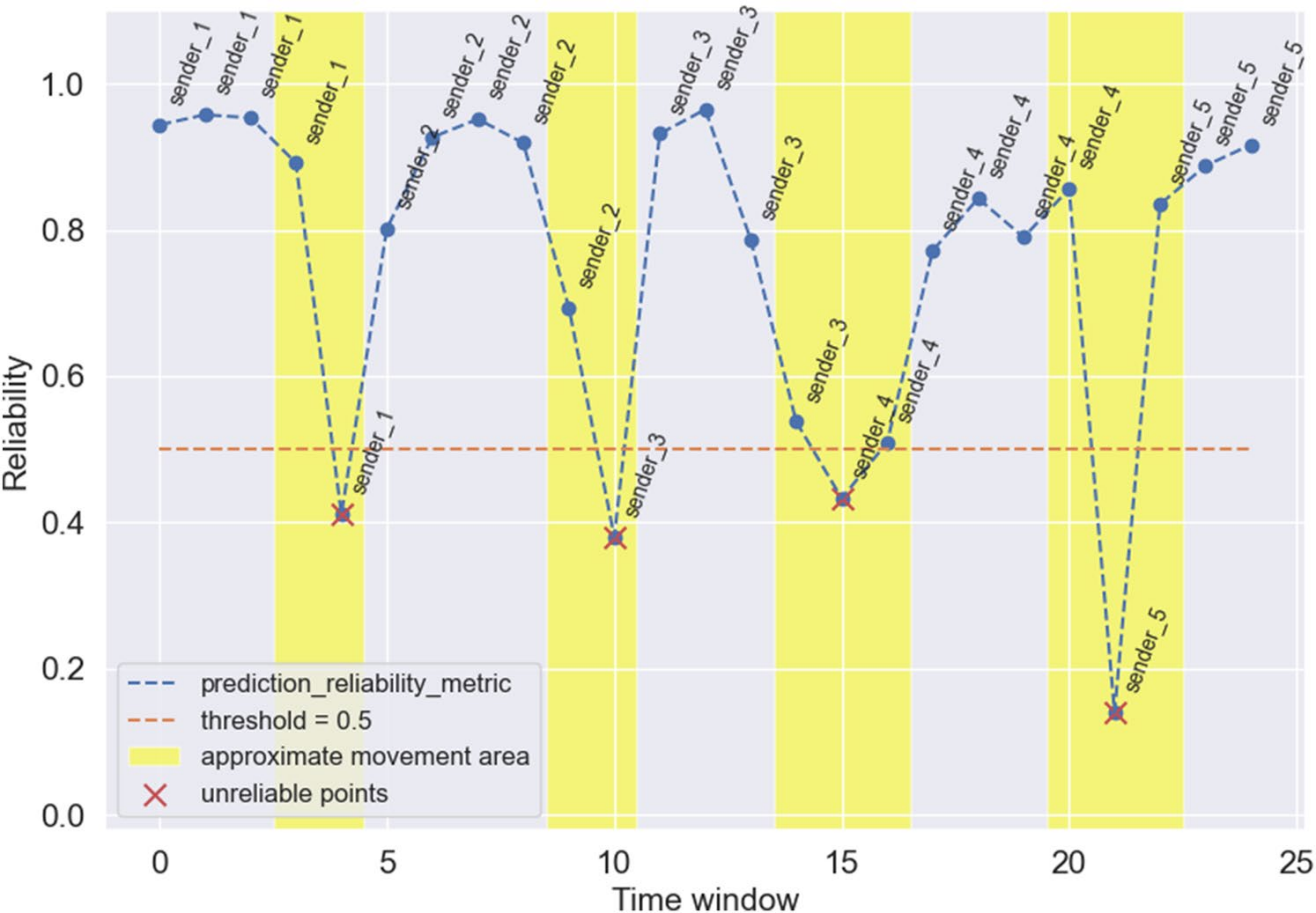
Filtering of unreliable proximity estimation results based on reliability metric over time.

In Figs. 6a,b,  and 7 we presented the filtering results for the three examined metrics (*activity_metric*, *rssi_metric* and *reliability_metric* respectively). In which examined time window we determined the closest stand. We highlighted the areas (with a margin) in which the movement occured. We also chose the values of thresholds (this value should be chosen according to how strictly we want to assess the reliability of data)—we set the threshold to 0.5 for *activity_metric* and *reliability_metric* and to 0.6 for *rssi_metric* (we used a higher value here, because the RSSI values which were used to calculate the metric are characterized by instability). Time windows, for which the value of a particular metric are higher than the threshold (for RSSI and activity-based metrics) or lower than the threshold (*reliability_metric*) are treated as unreliable and should be filtered (red crosses in the figures).

We can see that all of the metrics can be used to assess the reliability of the proximity detection (on the basis of movement detection). Even the *rssi_metric* (see Fig. 6a), which was calculated based on highly unstable Bluetooth signal strength exhibits a great power of movement detection in three out of four examined movement areas (movement in the third movement area was shifted to the no-movement area). It shows that even without using the readings from smartphone IMU we can efficiently detect the movement and filter unreliable predictions. The results obtained for the *activity_metric* (based on IMU readings, see Fig. 6b) are highly accurate and in all of the examined movement areas, the movement was detected (and in no-movement areas the values of the metric are close to zero suggesting no activity, which is true). If we use a metric based on activity and RSSI (see Fig. 7) we can significantly improve the accuracy of the RSSI-based metric itself and base our knowledge on both: changes in RSSI (an external information) and IMU sensors (an internal information).

## Discussion

The results show that the proposed system is highly effective and can be used in real applications. Due to the use of simple models for activity detection and simple methods for determining the value of RSSI-based metric, the solution is easy to implement, robust, memory and resource efficient and highly scalable. For these reasons, the solution is suitable for both offline and online analysis (with a slight delay—depending on the time window in which the analysis is performed).

In the case of offline analysis, it is possible to replace the simple activity detection model with a more advanced one (e.g. neural networks operating on raw data) in order to improve accuracy (it is worth noting that the accuracy of the created model is high too—with over 90% True Positive and True Negative rates). It is also possible to potentially improve the accuracy of the model using more comprehensive feature selection procedure (e.g., by using other filter-based methods: e.g., based on information gain and gini decrease measures, as well as wrapper-based and embedded-based methods).

The created system is flexible and can be customized: by choosing different values of the base in the softmax function, different values of the time window length, and values of the importance coefficient of the activity metric ($\alpha$ in Eq. 8) and threshold. Values of importance coefficients ($\alpha$ and $\gamma$ in Eq. 8) can be determined via simple optimization process, in which we aim to find such a value of one of the coefficients that allows us to obtain the maximum value of correlation between the reliability_metric and the labels representing the occurrence of a movement (we labelled our data manually based on the course of the experiment, see Fig. 5). We do not claim that this is the best approach, because one can also choose equal values of the coefficients to consider with equal power the results obtained through both primary metrics for simplicity. Such a solution draws fully from the advantages of both methods, but suffers just as much from their disadvantages as well (metric based on the ML algorithm prediction may potentially not detect a very steady walking, and RSSI-based metric can sometimes give poor predictions due to the susceptibility of the BLE signal to noise). Moreover, the decision regarding the importance (thus the coefficients’ values) of both basic metrics should be made by the creator of the real-world system powered by the proposed solution (such a person is best able to determine what kind of prediction quality is offered by e.g. the applied machine learning algorithm for activity prediction - because, of course, other algorithms than those presented in the article can be applied here, as long as they give analogous binary information on activity as an output). It is also possible to use only one of the base metrics tested (activity-based or RSSI-based) or to take only one of these metrics into account when creating a joint metric (to take only the activity prediction into account, set the importance to 1, and to take only the RSSI-based metric into account, set it to 0).

When a metric based only on RSSI is used for movement detection then there is no need to use IMU data and Machine Learning model and an even more computationally and memory efficient solution can be achieved (a mild trade-off between accuracy and efficiency, as the results for RSSI metric are also acceptable). Additionally, the great majority of operations can be conducted directly on smartphones (e.g. prediction of activity results and primary average value calculation), allowing partial distributed computing.

We used a relatively simple setup for our experiments (representing a simple exhibition space with stands). The configuration is basic due to the limited possibilities to perform measurements in the real environment during the Covid pandemic. Nevertheless, it can represent (at least to some extent) a typical room in an art gallery with a limited number of art pieces. Moreover, the scale of such a room is quite large, as the distance between the farthest anchors is over 11m. The approach can be extended to be used in larger environments. The measurements from embedded sensors during movements are independent of the external environment. However, the possibility of reception of RSSI frames from individual stands is limited by the distance from these stands. In the case of more complex configurations (and much larger environments), the receiver can only receive ADV frames from the closest stands. In this case, the average values of RSSI form stands, from which ADV frames cannot be received can be replaced with some very large value of RSSI (to point out that this value is actually NaN). Alternatively, the analysis can be performed only in the context of a segment (fragment) of the analyzed environment.

Another limitation is that (as already mentioned in “Data gathering”) in the case of smartphones, the ADV frame sending frequency is usually controlled by the operating system (it happens e.g. in iPhones). When the foreground mode of measuring application is used, a high frequency of ADV frames can be achieved, however in the background mode, some unexpected behaviours can occur. It is important to check how BLE works on the hardware platforms used in potential solutions. Of course, it is possible to obtain more control over the BLE operation, however it requires using dedicated BLE beacons instead of smartphones, which introduces additional costs and decreases practicality of a solution. However, the use of dedicated BLE beacons makes a lot of sense for fixed BLE beacon positions over long periods of time (e.g. in art galleries/ museums).

Moving people, crowded spaces and interference from other devices using the same radio frequencies impact the propagation of signal. It significantly limits the applicability of BLE solutions in general, however it is more prominent for distance estimation and localization and decreases the precision of these services. These factors have less impact on our solution because our goal is not to determine the exact location or distance estimation, but to determine the closest static point and filter out predictions for people moving between stands. Also, walking or movement can affect the RSSI readings. Therefore, our motion filtering solution can be used to filter out the results for distance estimation or localization (RSSI data can be aggregated only over a time window when the object remains in the same location—no movement is observed). The impact of disturbances on moving objects leads to a loss of precision, which further motivates to effectively discriminate between people who are moving and not. In addition, our system employs a kind of mitigation of the consequences associated with RSSI variability. It is the reason why we assign larger weights to closer static points for determining the RSSI-based activity metric. The signal at shorter distances is stronger, and there is less likelihood of excessive interference, since there is a smaller field between the dynamic and static object where potential interference can be introduced (e.g., by passing people).

The method presented in this article can be successfully used to filter out unreliable proximity detection results due to movement of the person for whom the proximity is detected. It is especially useful for the initial detection of the closest point (or with a slight extension—the closest points) due to its computational efficiency. To use it in practical applications, other methods can be added (e.g. ML-based proximity or distance estimation models) to further ensure that the positive proximity detection is not a false positive (it is truly a point of interest). In our future work, we will develop methods to increase the precision of proximity detection so that the solution is even more resistant to false detection of proximity. Here, we will consider, for example, filtering situations in which the person is standing in the middle of the room. For that purpose, we will build different experimental setups and design appropriate experiments reflecting as much possible situations as possible. We will also perform the experiments with more people. We also aim to create ML proximity detection and distance estimation models and incorporate them into our solution in the future.

## Conclusions

In this work we have presented the solution that can be used for cost-effective proximity detection and filtering of unreliable results. For that purpose we designed a system that utilizes BLE RSSI readings broadcasted by static points and IMU readings. As none of the benchmark datasets available online meets all of the necessary requirements (availability of static and dynamic activities, dependence of IMU readings on BLE frames arrivals, availability of raw sensory data), we gather all necessary data with a dedicated application. We use smartphones as senders and receivers of BLE frames and for gathering of sensor data (we use an embedded IMU to obtain readings from accelerometer, gyroscope and magnetometer). We have crafted three metrics that can be used for movement detection and filtering unreliable proximity detection results. We have created two base metrics: the first one based on RSSI readings (external world information) and the second one based on the predictions of created Activity Recognition Machine Learning model based on Gradient Boosted Decision Trees (internal object information). To create the efficient model, we have used one of the filter-based feature selection techniques and tested three ML models: Decision Tree and two ensemble models: Gradient Boosted Decision Trees and Random Forest. Using the base metrics, we have created a third metric—joint metric that combines the results of these two metrics.

It was shown that all of the proposed metrics can be used to assess the reliability of the proximity detection. It was proven that even without using the readings from smartphone IMU we can efficiently detect the movement and filter unreliable predictions (based only on RSSI readings). On the other hand, when we incorporate analysis of readings from IMU we can get very accurate movement detection and improve accuracy of our system. This way, we rely on both: changes in RSSI (an external information) and IMU sensors (an internal information).

The proposed system is flexible and can be easily customized by changing the values of the parameters used. Depending on the information at hand, reliability assessment can be based on the strength of the BLE signal or the readings from the IMU (or both of them). The created metrics do not require expensive calculations, thus can be used even in nearly real-time applications. Moreover, the great majority of operations can be conducted directly on smartphones, allowing for partial distributed computing. Additionally, the metrics are easy to interpret, even for a non-technical operators. Using smartphones (which are usually embedded with BLE and IMU modules and provide ML engines) to conduct the experiments results in potentially easy implementation, cost-efficiency and deployment of the solution in real-life applications in the areas of MICE industry, museums, art galleries and even industry. On the other hand, replacing smartphones with dedicated BLE devices may further improve accuracy of the solution (as these devices allow advanced calibration and control over sending frames).
