# Author Correction: Cost-effective filtering of unreliable proximity detection results based on BLE RSSI and IMU readings using smartphones

**DOI:** 10.1038/s41598-022-07289-y

**Published:** 2022-02-22

**Authors:** Katarzyna Filus, Sławomir Nowak, Joanna Domańska, Jakub Duda

**Affiliations:** 1 Institute of Theoretical and Applied Informatics, Polish Academy of Sciences, Bałtycka 5, 44-100 Gliwice, Poland; 2 Meetlify, Aleksandra Lubomirskiego 27/1, 31-509 Kraków, Poland

Correction to: *Scientific Reports* https://doi.org/10.1038/s41598-022-06201-y, published online 14 February 2022

The original version of this Article contained an error in Affiliation 2, which was incorrectly given as ‘Meetlify, Aleksandra Lubomirskiego 27/1, Katowice, 31-509, Poland’. The correct affiliation is listed below:

Meetlify, Aleksandra Lubomirskiego 27/1, Kraków, 31-509, Poland

The original Article has been corrected.

